# Navigating toward resilient and inclusive seed systems

**DOI:** 10.1073/pnas.2218777120

**Published:** 2023-03-27

**Authors:** Ola T. Westengen, Sarah Paule Dalle, Teshome Hunduma Mulesa

**Affiliations:** ^a^Department of International Environment and Development Studies, Faculty of Landscape and Society, Norwegian University of Life Sciences, 1430 Ås, Norway

**Keywords:** seed systems, seed security, genetic resources

## Abstract

Food systems face new climatic and socioecological challenges and farmers need a diversity of new plant varieties to respond to these. While plant breeding is important, institutional innovations in *seed systems* are critical to ensure that new traits and varieties make their way into farmers’ fields. This *Perspective* reviews the state of knowledge on seed system development, outlining insights emerging from the literature that can help navigate the way forward. We synthesize evidence on the contributions and limitations of the different actors, activities, and institutions pertaining to all seed systems smallholder farmers use, formal and informal. To do so, we structure our analysis on three functions—variety development and management, seed production, and seed dissemination—and two contextual factors—seed governance and food system drivers—that can be used to describe any seed system. Our review reveals the strengths and weaknesses of the activities of different actors along the entire chain of functions and demonstrates the multifaceted efforts to strengthen seed systems. We document that a new agenda for seed system development is taking root, based on the view that formal and farmers’ seed systems are complementary. Because needs differ from crop to crop, farmer to farmer, and between agroecological and food system contexts, a variety of pathways are needed to ensure farmers’ seed security. While the complexity of seed systems eludes a simple roadmap, we conclude by planting a “signpost” with principles to guide efforts to develop resilient and inclusive seed systems.

Crop diversity and new plant varieties are essential in food system transformation. Conservation of genetic resources, base-broadening, and breeding is crucial for introducing new traits for tolerance to multiple stressors, not least those caused by climate change (1, 2). But breeding alone is not enough. New traits and varieties must make their way into farmers’ fields, and there is increasing recognition that alongside technical innovations, institutional innovations in *seed systems* are also key to increasing farmers’ access to needed crop diversity (3–5).

This Perspective reviews the state of knowledge on seed system used by smallholder farmers in low- and middle-income countries, outlining insights that can help navigate toward more resilient and inclusive seed system development. In the last 15 y, there have been increased investments in seed system development in many countries in the Global South. However, there is a lack of agreement on the best way to achieve national seed systems that contribute to food security, climate adaptation, and poverty alleviation, as well as social, environmental, and economic sustainability. Debates related to seeds have been hotly contested in both national and international arenas, pitting agendas of agricultural modernization against those of food sovereignty (6). It has also been reported that actors often lack understanding of the broader seed systems in which they intervene (7, 8). At the same time, efforts have been made to bridge this divide and find ways forward. Research has deepened our understanding of the functioning of seed systems and the implications for meeting farmers’ needs and demands while new policy approaches promoting pluralism and mechanisms for regulatory flexibility have been rolled out. In short, a new agenda for seed system development seems to be taking root (9).

## Seed Systems: Theory and Practice

Seed systems are understood as the actors, activities, and institutions involved in the maintenance of crop diversity, plant breeding and selection, seed production and dissemination (10). In short, they are the systems that make seed available to farmers. In theory, a well-functioning seed system will ensure *seed security* for all farmers, i.e., that “men and women within the household have sufficient access to adequate quantities of good quality seed and planting materials of preferred crop varieties at all times in both good and bad cropping seasons” (11). In reality, this is rarely the case. Seed systems can be disrupted by both acute stresses such as conflicts and disasters, and chronic problems relating to social inequalities, inefficiencies, lack of coordination between actors, or inappropriate policies and regulatory frameworks (5, 12, 13). Thus, it is important to recognize that seed systems are influenced by the broader context in which they operate.

Generally, it has been common to distinguish between different types of seed systems which vary in terms of the actors involved and their roles, the diversity and volumes of seed produced, as well as in their governance mechanisms. The *informal system*—also called traditional, local or farmers’ seed system—refers to the practices and institutions that are involved in farmers’ on-farm management of crop diversity, and in their access to seed through own production, farmer-to-farmer exchange, and local markets. These practices are largely mediated by social rules and norms that have evolved over time and are closely linked to local cultures and traditions. The *formal seed system* is understood as the development, distribution, and sale of certified seeds of “improved” varieties in registered outlets. It usually covers only a few crops with higher commercial value. This system is generally governed by national policies and legal frameworks defining variety release, seed certification and phytosanitary controls (10). Finally, a third type of seed system—the *intermediate seed system* is also increasingly recognized, referring to individuals or organized farmers that produce and sell seed not sufficiently covered by the formal seed system, often following simplified certification schemes (10, 14).

The distinction made between these seed systems, however, is problematic on at least two counts. First, evidence shows that there are many linkages and interdependencies between formal and informal seed systems (15). In the theory of change underpinning the Green Revolution, informal seed systems were expected to gradually be replaced by formal seed systems (16–18), but for many crops and parts of the world, this has not, or only partially, happened. Studies and experience show that farmers often source seeds from all the seed system types mentioned above (5, 15, 19). Second, the terms themselves are imprecise and can reinforce misconceptions. For instance, the term “informal” can misleadingly imply that such seed systems are not rule-governed, while “local” suggests that seed only circulates at limited geographic scales, while there is ample evidence to the contrary (20–23).

**Fig. 1.**
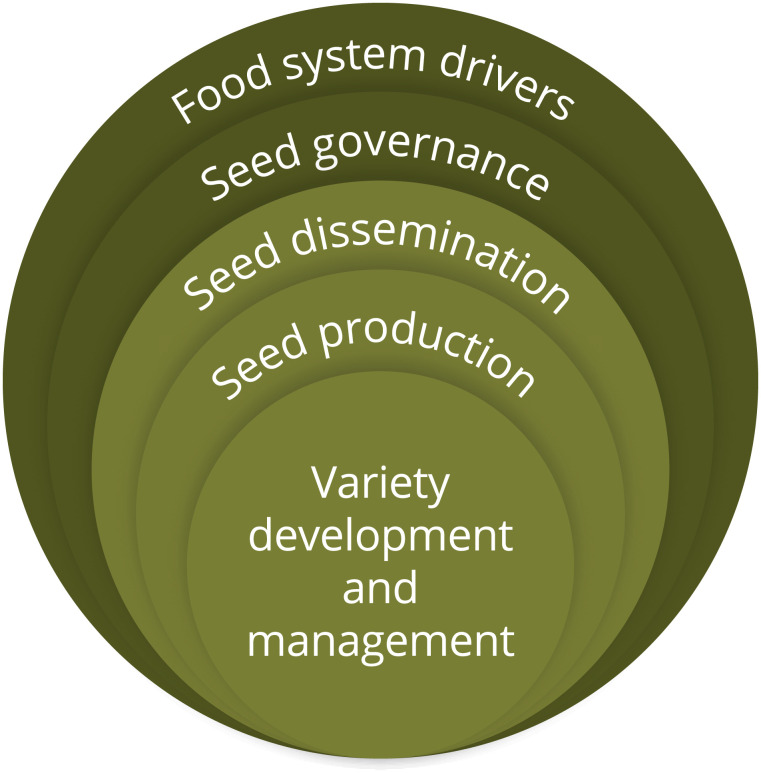
Conceptual framework with key functions and contexts for seed system development.

In this article, we discuss seed systems in a holistic way, drawing on literature and insights pertaining to all “seed systems farmers use” (5). To do so, we structure our analysis on five factors (Fig. 1) that can be used to describe any seed system (24–26). Variety development and management, seed production and seed dissemination are three *key functions* that seed systems must deliver to be effective. Seed governance and food system drivers are *contextual factors* that influence how the first three functions operate. We discuss each of these functions and contexts, synthesizing evidence on the contributions and limitations of different actors, activities, and institutions, and highlight promising ways forward.

## Variety Development and Management

Variety development and management, including the evolution, breeding, maintenance, and conservation of varieties, is a fundamental seed system function in which both farmers and professional breeders play important roles. For millennia, farmers’ seed systems have created and maintained crop diversity that provides essential functions for farm households (27–30). For many crops, “landraces”—or farmers’/traditional/local varieties—are the major source of seeds planted by small-holder farmers (31). Farmers also experiment actively with genetic materials to create new diversity, using a range of different selection practices (32), sometimes hybridizing (or “creolizing”) local and improved varieties (33, 34), and allowing (deliberately or not) geneflow between wild relatives and cultivars (35, 36). As such, farmers’ cultivation and innovation are fundamental to maintaining genetic diversity, as well as evolving and adapting crop varieties to new conditions and needs (37). At the same time, farmers’ decisions about what crops and varieties to plant are influenced by agroecological, climatic, demographic, economic and cultural factors (38–41). This dynamic nature of farmers’ variety management introduces the potential for increases or losses of genetic diversity (genetic erosion) as well as gene flow into traditional varieties from improved varieties, including transgenic varieties (42, 43). With the modernization of agriculture, displacement of landraces by improved varieties is recognized as the most common cause of genetic erosion, though other drivers including shifts in labor availability, markets, and environmental conditions, including climate change, also play a role (39).

There have been different responses to the threat of genetic erosion, including both efforts to strengthen farmers’ maintenance of crop diversity on-farm (in situ) and as well as the collection, and conservation in genebanks (ex situ). On-farm approaches aim to promote conservation by addressing key constraints that limit farmers’ use of crop diversity. This can include increasing availability of and access to crop diversity and related information, creating or promoting markets for neglected/underused varieties, improving local materials through participatory selection or breeding to remove undesirable traits, building or strengthening local institutions and farmer leadership, as well as promoting changes in policies (31). The advantage of on-farm conservation is that varieties circulating in farmers’ seed systems continue to be adapted to new conditions, but at the cost of losing adaptation to those of the past (44). In contrast, ex situ conservation in national and international genebanks is considered the most certain way to conserve the genetic makeup of a variety at a particular point in time. Consequently, genebank accessions have been used to reintroduce lost diversity to localities where landraces have been lost in the field (45). But as the number of genebanks has grown globally it has become increasingly clear that not only diversity in situ, but also ex situ collections are vulnerable to genetic erosion due to funding constraints and lack of technical capacity for regeneration and other vital germplasm management (39, 46, 47). Both ex situ and in situ conservation approaches have their advantages and limitations, justifying the need for an integrated approach to conservation and management of crop gene pools.

Plant breeding is essential to adapt agriculture to new conditions and needs. One of the advantages of formal plant breeding is its access to a wide gene pool—facilitated by international and national genebank collections—as well as increasingly sophisticated breeding methods. The threat to crop cultivation posed by climate change is a good example of the importance of access to genes and traits from outside the climate envelope to which currently grown varieties are adapted (48). However, a typical challenge has been addressing farmers’ needs and preferences in diverse socioecological conditions. Farmers are able to select varieties adapted to their local conditions, preferences and needs, and studies show that the lack of farmers’ participation can explain the low adoption rate for some of the conventionally bred improved varieties (49, 50). To address this, decentralized and participatory approaches to plant breeding and variety selection have emerged since the 1980s as an alternative and complement to conventional plant breeding (51–54). There is no contradiction in combining participatory plant breeding with cutting-edge molecular approaches to plant breeding such as marker-assisted selection and gene editing; many of the current crop improvement programs of, for example, the CGIAR integrate the two. While initiatives may operate with different theories of change and normative ideas about agricultural development, there seems to be growing recognition of the need to become more demand-oriented. Tools are developed and deployed to make “product profiles” that can make breeding programs more gender-responsive (55). Promising results are coming out of the use of the citizen science “triadic comparisons of technologies” approach involving large numbers of farmers in the on-farm evaluation of sets of three varieties of a given crop species which, combined with use of digital tools, enables both local adaptation and a scale of reach not seen in earlier participatory plant breeding (56–58).

## Seed Production

Seed production is key to ensuring the availability of sufficient quantities and qualities of seed of diverse crop varieties. Different models for organizing seed production have developed over time, depending on the crop or variety, its use, commercial value, and the quality standards used. These include farmers’ own seed production, centralized certified seed production, and, more recently, different models for decentralized seed production.

Farmers’ knowledge and practices have played a crucial role in local seed production and storage, and in most Global South countries, farmers dominate in terms of the volume of seeds produced and the number of crops and varieties covered, compared to the formal seed system (20). This is also the case for important self-pollinating crops in countries in the GlobalNorth such as wheat in North America (59). There are many examples of traditional seed storage technologies as well as norms, and practices to ensure seed quality during the pre- and postharvest seed processing stage (60, 61). One advantage of on-farm seed production and storage is that seed is readily available for farmers at planting time (5, 15), and is much less costly than certified seed (59, 62). However, farmers’ seed production and storage practices often differ little from those for grain, and although farmers have knowledge and skills in seed selection, treatment, and storage, their seed production practices often have shortcomings (63). Common issues include control of weed contamination and disease transmission (especially in vegetatively propagated seeds), timing of seed harvest, drying to acceptable moisture content, proper seed storage, and regular selection to avoid degeneration in local varieties (64–66). That said, farmers often perceive seed quality differently than formal seed system experts depending on quality attributes (e.g., size, color, and taste) and value considered (e.g., productivity vs. tastiness and versatility of the seed) (67), and may not always prefer nor be able or willing to pay the higher price for certified seed (8). Nonetheless, seed quality is an important factor in determining yield and other agronomic performance in crop production and is a common challenge to farmers’ seed security (5, 12).

While farm-saved seed has been the backbone of seed use for millennia, models for centrally organized, specialized seed production was introduced in many countries in the Global South in the 1950s. Focused on the production of certified seed of formally released improved varieties, these efforts have been led by private commercial, public, and semipublic companies. Private seed companies mainly concentrate on crops with a high multiplication factor (i.e., high net yield of seed per seeding rate) which are more profitable, whereas most public seed producers are dedicated to certified seed production of major food security crops less attractive to the private sector. Aimed at reaching national, regional and/or international seed markets, private and parastatal companies produce seed on large farms and/or contract small- and medium-scale farmers as out-growers to scale up production. They also interact with agencies responsible for producing Early Generation Seed (EGS) (*breeder seed* and *foundation/basic seed*) as well as the seed certification authorities. This model has clear advantages for seed production of crops or varieties that require advanced management and intensive labor (e.g., hybrid varieties) or biennial or perennial crops whose seed production competes with food production, making it difficult for smallholder farmers to dedicate land for this purpose (e.g., carrots or fruit/nut trees). This said, certified seed production also faces several challenges. Common issues include insufficient availability of EGS (12, 68), economic and technical barriers such as high investment capital need, high multiplication cost of hybrid seeds, as well as competition for commercial producers from farmers recycling open-pollinated varieties (69). Even when multiplication works well, producers have problems with the transport and storage of seeds in remote areas. Some solutions have been proposed, such as revising the roles of public agricultural research institutes and parastatals to focus on the supply of EGS of diverse crops and varieties, leaving seed multiplication and dissemination to seed producers and distributors, thereby strengthening the interface between public improved variety/germplasm providers and seed producers (70–72). Nonetheless, seed production by public and private companies remains limited in the Global South (73, 74), covering less than 20% of the seed demand for most food crops in many countries (5, 10).

In response to some of the challenges linked to certified seed production, more decentralized models have been promoted since the 1990s. These include initiatives that build the capacity of local actors to produce seed and increase its availability, though they vary in terms of the crops and varieties targeted, the actors involved and their stated goals. For example, community seed banks are local institutions supported mainly by Non-governmental Organizations (NGOs) to multiply and distribute seeds of local crops and varieties for conservation, to increase local availability and serve as a reserve in times of stress (75–77). In contrast to such non-profit organizations, decentralized seed producer cooperatives or local seed businesses have a market orientation. Typically owned by farmers, these are small to medium-scale seed enterprises that collaborate with formal sector institutions, including research, extension services, and NGOs to produce seed of local and improved varieties for marketing at affordable prices (78–81). Several assessments have observed that the embeddedness of such decentralized seed production initiatives in local community organizations contributes to their effectiveness and performance (82), although questions are raised about the long-term sustainability of the local institutions once external support is withdrawn (83, 84), but recent assessments show profitability and sustainability of local market-oriented seed production initiatives (85, 86).

## Seed Dissemination

Seed dissemination refers to the distribution of seed from the place of production and/or storage to its acquisition by farmers. The specific channels used by a given farmer vary according to what sources are available in his or her locality, the diversity and types of crops and varieties he or she cultivates, as well as a host of economic, social, cultural and political variables that influence decision-making at both individual and household levels (4, 5, 10). Here we discuss the strengths and weaknesses of different dissemination channels, as well as new innovations that hold promise to improve access to seed by farmers.

Farm-saved seeds are the primary source of planting material for many smallholder farmers for local varieties with specific cultural, spiritual or agroecological values (87) as well as recycled improved varieties (88). Farm-saving of seeds have several benefits to smallholders, including reduced cash expenditure to acquire seeds and secured availability of seeds at planting time. Moreover, farmers are generally more familiar with the agronomic, post-harvest and consumption attributes of their own-saved seeds and may prefer them for this reason (89). That said, most households do not produce enough seed to meet all of their seed requirements, and use other sources to increase seed volume, replace poor quality seed, acquire seed that they lack knowledge, skill or adequate conditions to produce (e.g., exotic vegetables) or store (e.g., many legumes), or to obtain new varieties with desired traits (90). It is also important to recognize that some households may not produce their own seed at all, due to land scarcity or other reasons (91).

Recent research has revealed that local markets play a more critical role in seed dissemination in the Global South than previously understood. According to a comprehensive study of seed sources farmers used for 40 crops in six countries in postdisaster situations, about 51% of the seeds obtained from informal sources came from local markets, exceeding farmers’ own saved seeds by 20% (5). This is not limited to local varieties. According to a recent study on yellow bean seed trade in Tanzania, 60% of the seeds acquired by farmers were improved varieties bought from local traders, two of which had only been released 1 to 3 y before, demonstrating rapid dissemination through trade networks (23).

Social networks also contribute to seed dissemination and the functioning of seed systems, though their importance varies by crop and context (20). In these networks seeds are shared among relatives, neighbors and friends either as gifts or exchanged (e.g., for labor, another crop or variety) or purchased for cash (92, 93). Social network analysis (94), have shed light on the functioning of these exchanges, demonstrating for example how nodal farmers play a key role in the diffusion of new varieties of barley in Syria (95) and Ethiopia (96), and in provisioning traditional varieties and maintaining crop genetic diversity on-farm in Nepal and China (97, 98). It is also important to recognize that social networks are subject to social, economic, and cultural dynamics. Seed flows may follow ethnolinguistic lines, exclude certain individuals or households, or break down where social relations or trust are eroded and cropping systems are disrupted (20, 99–101). Seed exchange networks can also contribute to the spread of crop diseases by disseminating infected planting materials (22, 102). Indeed, personal relationships in social and kin networks are often strong social institutions based on solidarity and reciprocity that may provide sufficient trust in seed quality between providers and receivers (103), but this should be considered an empirical question, rather than assuming a priori that social networks always “ensure ready, egalitarian access to seed” (20).

Seed channels typically associated with the formal seed system include agricultural research institutions, parastatal companies, government extension services, agrodealers, and large farmers’ cooperatives/unions. Common challenges in public systems include high overhead costs and inefficiencies that often lead to late delivery of seed, insufficient supply, or low seed quality (83, 104). This is further complicated by the fact that in some countries, parastatals have monopolies over certified seed dissemination for political reasons (72). Private seed companies distribute seeds through their marketing outlets, such as agrodealers and cooperatives. Although this is considered more cost-efficient than seed dissemination by parastatals, it is expensive for poor farmers and limited to few crops and varieties, e.g., hybrids (105). Agrodealers also tend to have poor coverage in more remote areas (106). Efforts to address these challenges are ongoing, especially in Africa, with investments generally targeting private sector channels. For instance, after decades of state monopoly in seed supply, the Ethiopian government in 2011 introduced direct seed marketing to farmers through certified individuals and cooperatives (107). Other approaches aim to build directly on linkages with farmers’ seed systems, for example by using model or nodal farmers to disseminate new varieties and seeds from agricultural research institutions (108), or allowing out-grower farmers to legally retain a portion of the seed production for faster dissemination through social networks (13).

Seed dissemination by community-based groups, NGOs, and humanitarian organizations generally play a smaller role than the other sources described above. Since the 1970s, seed relief programs run by governments or NGOs have become increasingly common, typically distributing free—often certified—seeds during humanitarian crises (109, 110). In the last two decades, analyses of farmers’ seed security in emergency contexts have revealed a number of weaknesses in seed relief efforts (8) leading to calls for better assessment of seed security needs to design more targeted responses (11, 111, 112). One response has been to shift from direct seed distribution to the use of vouchers and cash transfers to address households’ lack of purchasing power, identified in many assessments to be a key constraint (113, 114). Another message from the literature on seed security in humanitarian contexts is as mentioned above, that traders in informal/local markets supply a considerable share of the seeds used and therefore merit more focus as potential partners in seed aid and seed system development (5, 115). Apart from seed aid, many decentralized seed producer groups discussed in the previous section also engage in seed dissemination, selling seed directly to local community members. Community seed banks, local seed businesses and seed producer groups can fill a niche by offering access to good quality seeds of crops and varieties not covered by other seed sources (79, 116). Furthermore, they help make seed more accessible to resource-poor farmers, by offering seed on loan to their members that can be repaid in kind at harvest or for purchase at more affordable prices than certified seed. Promising results are reported for these intermediate approaches to seed dissemination, but more independent empirical research is needed into their performance, scalability, and sustainability.

## Seed Governance

Governance refers to the way societies handle issues of coordination and conflict regarding the use of resources (117). A range of formal and informal rules govern crop diversity and seeds: international biodiversity agreements and their access and benefit sharing (ABS) provisions govern access to and use of genetic resources; intellectual property rights (IPR) govern the ownership and use of new plant varieties at both international and national levels (118–120); and national seed laws and regulations govern the registration of varieties and seed producers and distributors, as well as seed quality control (78, 121–123). In addition to such formal rules and regulations, social norms, customary laws, principles, and beliefs influence farmers’ practices in seed saving, exchange, and use (124–126), as well as those of other seed system actors (127, 128). The multiple layers of policies, laws, regulations, and norms governing seed systems influence seed system outcomes in significant ways.

One of the central governance issues affecting variety development and management concerns the “ownership” of crop diversity, which affects farmers’, breeders’, and other seed system actors’ access to genetic resources. It is also one of the most challenging, as there are fundamentally different views about what, if any, part of the diversity can be owned, and by whom. Whereas crop diversity historically has been governed as a public good, new varieties released by plant breeders are today commonly governed as private goods through various types of IPR protection. Legal access restriction to crop diversity arguably started when the US congress passed the Plant Patent Act in 1930 (129). This was followed by adoption of legal IPR protection in other countries, a trend that was accelerated by the Trade Related Aspects of Intellectual Property Rights Agreement (TRIPS). Adopted in 1994, TRIPS requires all World Trade Organization member countries to implement either patent protection or an effective *sui generis* (of its own kind) IPR system for new plant varieties (130). While IPR aims to protect the rights of breeders and companies who invest in developing new varieties, international ABS regimes aim to protect the rights of states and local guardians of crop diversity whose genetic resources are used in innovation processes. In 1991, a Food and Agriculture Organization of the United Nations (FAO) resolution, later cemented in the Convention on Biological Diversity (CBD) in 1992, made the historical principle defining Plant Genetic Resources for Food and Agriculture (PGRFA) as the “common heritage of mankind” conditional on the “sovereignty of the states over their plant genetic resources” (131). This entails that states now can restrict access to what was earlier a “PGRFA commons”. PGRFA falling under the mandate of the CBD are normally governed by national ABS laws and bilateral material transfer agreements. And while the International Treaty on Plant Genetic Resources for Food and Agriculture (ITPGRFA) later re-established a kind of commons-based governance for 64 of some of the most important agricultural and forage crops through its Multilateral System, users of crop diversity today must navigate between restrictions posed by ABS laws on the one hand and IPR laws on the other (132, 133). Currently, the ABS and IPR enclosures in the PGRFA commons makes it challenging for breeders and farmers to access the genes and traits useful in the development of varieties adapted to new climatic conditions and other agroecological and social demands.

The international debate about access to crop diversity is so heated that it has been called “the seed wars” (134). Many scholars and activists argue for a fundamental reorientation from protecting breeders’ and companies’ IPRs to protecting farmers’ rights to save, use, exchange and sell seeds as recognized in the ITPGRFA and in the United Nations Declaration on the Rights of Peasants (135–138). Some take an “ownership approach” to farmers’ and peasants’ rights, formulating them in direct opposition to the breeders’ rights protected by IPRs, while others take a “stewardship approach”, emphasizing that crop diversity in all its forms should be governed as a public good (131, 139). Others, thinking outside the box of international biodiversity and IPR treaties and instruments, argue for “commoning” all forms of crop diversity management through collaborative network approaches to seed governance (140–142). Similarly, some breeders and organizations promote “Open Source Seed” pledges on new varieties as a means to maintain open access to germplasm (143, 144).

In the last years, there have been increased efforts to introduce regulatory reforms and innovations in seed governance, which arguably have shown more promise for getting beyond the divides than the discussions in the international crop diversity governance forums discussed above. One area where regulatory flexibility has been introduced is in the process for variety registration and release. Seed laws typically have not allowed for the registration, dissemination, and, in particular, commercial sale of farmers’ varieties (121). Before a new variety can be released it typically has to meet standards for distinctiveness, uniformity, and stability (DUS test) as well as prove that it has value for cultivation and use (VCU test) in multilocation national performance trials over two to three seasons. In order to speed up the process and allow more flexibility in terms of what types of varieties can be registered, some countries have introduced measures to either relax DUS and VCU testing requirements or adopted alternative seed catalogues for farmers’ varieties (e.g., Ethiopia, Benin, India, Nepal, Brazil, Peru) (123). The European Union’s variety registration system has also recently been relaxed to allow for registration and marketing of “conservation varieties” and “organic heterogenous material” (145), an example of how it is increasingly recognized also in the Global North that rigid formal system approaches are ripe for reform.

Reforms have also been proposed to improve efficiency of seed quality control by introducing more voluntary and trust-based approaches (17, 71, 105, 146, 147). Certification is the formal institution created to ensure that the seeds farmers buy are of high quality, meeting standards for genetic purity, physical purity, and germination, but is often hard to implement in the organizational and economic context of agriculture in the Global South. To address this, different alternative schemes have been suggested, tested, and scaled up for several crops in different countries. The best-known example of an alternative quality assurance system is the Quality Declared Seed (QDS) scheme introduced by FAO in 1993 (148, 149). This system allows for production of a QDS class of seeds in which certification authorities control a smaller subset of seed batches with less rigorous standards than in regular certification. The QDS scheme has also been revised to allow for production and sale of farmers’ varieties (150), but the scale of QDS production and dissemination remains low in many countries due to limited added value for both suppliers and buyers especially when sales are only legally permitted in nearby localities (123, 151).

These questions about who can produce and sell what and where are also regulated by national seed laws which typically require registration or licensing of seed producers and dealers. A few national seed laws explicitly recognize and allow for registration of informal seed dealers (e.g., India and Vietnam), while others leave some room for flexibility by exempting smallholders engaged in customary seed sale from requirements to register (e.g., Peru and Brazil, Zimbabwe) (115, 121). Scholars and practitioners promoting an increased role for local market traders in seed security efforts recognize the need to focus on how to ensure variety and seed quality in such sales (115). In long term seed system development efforts, it is important that increased regulatory flexibility in the informal system are balanced with appropriate institutional measures for protecting farmers from counterfeit varieties or poor-quality seeds when buying seeds outside their traditional social spheres of trust.

The examples of regulatory flexibility reviewed above represent modifications of existing governance arrangements that aim to improve the efficiency of key seed system functions, while also opening space for greater participation of smallholder farmers. In addition to this, there are efforts to recognize and promote the co-existence of seed systems through broader reforms to seed policy. The FAO voluntary guidelines for national seed policy formulation published in 2015 stated that “*a national seed policy is a statement of principles that guides government action and explains the roles of relevant stakeholders in the coordination, structure, functioning and development of the seed system comprising both formal and informal sectors*” (152, p. 4). In line with this notion of complementarity between formal and farmers’ seed systems, some African countries have adopted what is referred to as pluralistic or integrated seed system development policies (e.g., Ethiopia, Uganda) which explicitly identify strategies to strengthen formal, farmers’ and intermediate seed systems (153, 154). Many of the innovative approaches described in the functions sections above are indeed promising offspring of this agenda.

## Food System Drivers

Seed systems are subsystems of larger food systems. During the last decade or so, the food system concept has become the most important framing of food and agricultural development questions both in scholarly and policy debates (155). Food system frameworks depict how food systems produce food security, health, as well as other social, economic, and environmental outcomes, which feedback as drivers of food system change. Here, we focus on two aspects important for seed system development that food system frameworks help us see more clearly: the importance of understanding heterogenous demand for crop diversity and the influence of political–economic drivers.

Farmers’ demand for crop diversity is highly heterogenous. Food system frameworks show how food supply is inextricably linked to households’ demand as well as the setting in which the exchange or transaction takes place (referred to as the “food environment”) and how these dynamics are affected by factors across a range of scales from the local to the global. The low adoption of improved varieties of many crops in the Global South is due to a mix of such systemic factors. The choice of seed depends, *inter alia*, on the economic importance of farming relative to other livelihood strategies, whether the crop is for sale or home consumption, physical and economic access to the marketplace, behavioral and cultural context of the household, the human security situation and the political and economic context (5, 19, 156, 157). Adoption studies are often trapped in the linear theory of change underpinning many seed supply efforts and new systemic and contextualized approaches to understand seed demand are needed (158, 159). Taking a demand-centered rather than a supply-centered seed system development perspective will in most cases show that a diversity of needs requires a diversity of seed system approaches.

Political–economic drivers are evident with the globalization of food systems over the last decades, which also includes seed systems. Seeds have become big business. The four largest multinational companies in seed trade today control about 60% of the ~50 billion USD annual global commercial seed market (160, 161). This is the result of several waves of consolidation in the seed and agrochemical industry with acquisitions of smaller seed companies (and their IPRs) and mergers between the larger ones. The large private actors have the power not only to shape markets, but also science and innovation agendas as well as policy frameworks. Private sector research and development typically focuses on the most profitable crops and breeding technologies, such as genetically modified maize and soy and associated agrochemical inputs. In terms of policy influence, free trade and regional trade agreements commonly include requirements for implementation of IPR protection and harmonization of seed regulations to attract commercial interests. When influential donors and governments support this private sector centered development agenda in countries where farmers’ seed systems dominate, the mismatch between seed regulations and customary seed exchange practices often leads to tension and conflict (162, 163). Unintended negative consequences for crop diversity conservation might arise from the complex system interplay between seed laws and farmers’ variety management when conventional and transgenic improved varieties are grown side-by-side in centers of crop diversity (42, 43). Concerns about potential negative environmental and social equity outcomes have led food system analysts to call for better international and national regulation of corporate power concentration (160, 164). In addition, supporting public breeding programs for commercially less-interesting crops and creating enabling institutional environments for the range of seed systems farmers use is important for balancing out some of the transformative impacts of globalization. These political economic dynamics form the backdrop of food systems, which both shape and constrain efforts to improve the functioning and governance of seed systems described above.

## Signpost for Resilient and Inclusive Seed Systems

In this *Perspective* we have provided an overview of approaches to support the seed systems farmers use along the entire chain of functions from variety development and management to seed production and dissemination. We have documented that a new agenda for seed system development is taking root around the world, based on the view that formal and farmers’ seed systems are complementary. This new approach is informed by empirical research into farmers’ seed use, revealing that despite decades of large investment in formal seed system development, farmers in many countries in the Global South largely rely on farmers’ seed systems to source seeds for most of their crops. It is also informed by experiences of different seed system actors to reach farmers in alternative ways than as merely commercial end-users of technology. Because needs differ from crop to crop, farmer to farmer and between agroecological and food system contexts, a variety of pathways are needed to reach this goal. Here we plant a signpost with some general principles for the way ahead.

## Do No Harm

Farmers’ seed systems are crucial in the livelihoods of millions of farmers, as well as for long term food system sustainability. Most of the seed farmers use in the Global South are sourced from own harvest or exchanged or traded in social networks or informal markets (5). While these seed systems are not in and of themselves equally accessible to all farmers, they form the backbone of the seed supply in many countries (20). The first principle for all seed system development efforts should therefore be to “do no harm” to these systems, but rather to build on them. Contrary to common belief, there is no conflict between this principle and the breeding and release of new varieties by formal systems; farmers’ seed systems seldom only move local varieties (e.g., landraces) but also new varieties originating from breeding programs. There is also not necessarily a conflict between this principle and programs and policies supporting formal seed system development; farmers often use different channels for different crops and draw on the strengths of the different systems. However, seed policies and laws meant to promote formal seed system development can have negative spill-over effects on farmers’ seed systems if they outlaw customary practices such as seed-saving and exchange. Integrated seed system development has gained ground in national seed policies and regulations over the last years, purportedly supporting coexistence between seed systems. To move from recognition in policy documents to implementation on the ground, funding and political will must follow.

## Diversity

Farmers need access to a diversity of crop species and varieties and this principle should guide the management and governance of all seed system functions. Too often the maintenance of diversity is considered a goal only in programs for crop diversity conservation, but not in variety development and supply. However, crop diversity is associated with livelihood and food system resilience both at the farm level (165) and at the national level (166). And as we have shown in the preceding sections, this is not only a question about biological diversity, but also in terms of institutions and actors involved throughout the seed system. A diversity of seed sources is needed to meet the needs of different kinds of farmers in heterogenous food system contexts (4). It is thus important to maintain and promote diversity of both crops and actors and actively counteract the diversity bottlenecks that can be caused by power concentration at different stages in the system. A promising approach to ensure coordination and maintain focus on overarching public good goals is the formation of collaborative networks or multistakeholder platforms with the participation of public institutions, NGOs, seed companies, farmer groups, and seed entrepreneurs (121, 140).

## Seed Security

The seed security perspective used to assess farmers’ access to preferred varieties of seeds in humanitarian contexts should also guide long-term seed system development. The seed system functions analyzed here are processes whose effects can be assessed in terms of seed security outcomes for farmers. The dimensions of seed security correspond to the four commonly recognized dimensions of food security: availability, accessibility, quality, and stability. In terms of seed security, quality is both a question of varietal suitability, and genetic, physical, and physiological properties and stability is question both of farmers’ stable access over time and the resilience of the system to recover from shocks and stress (11). The latter is a crucial element to consider for seed system projects in conflict- and disaster-prone areas where seed systems must be able to adapt to and recover from shocks also when formal systems stop functioning. Recently, two additional dimensions are proposed for food security: sustainability and agency (167). We suggest that these two new dimensions are also useful additions to the seed security framework, drawing attention to the need for seed systems to maintain the biological basis for long term functioning and the importance of being attentive to how power is distributed in the system. Seed system security assessments including all these dimensions will provide a solid evidence basis for truly resilient and inclusive seed system development going forward.

## Data Availability

There are no data underlying this work.
